# Molecular Signatures of Hemagglutinin Stem-Directed Heterosubtypic Human Neutralizing Antibodies against Influenza A Viruses

**DOI:** 10.1371/journal.ppat.1004103

**Published:** 2014-05-01

**Authors:** Yuval Avnir, Aimee S. Tallarico, Quan Zhu, Andrew S. Bennett, Gene Connelly, Jared Sheehan, Jianhua Sui, Amr Fahmy, Chiung-yu Huang, Greg Cadwell, Laurie A. Bankston, Andrew T. McGuire, Leonidas Stamatatos, Gerhard Wagner, Robert C. Liddington, Wayne A. Marasco

**Affiliations:** 1 Department of Cancer Immunology & AIDS, Dana-Farber Cancer Institute, Department of Medicine, Harvard Medical School, Boston, Massachusetts, United States of America; 2 Department of Biological Chemistry and Molecular Pharmacology, Harvard Medical School, Boston, Massachusetts, United States of America; 3 Biostatistics Research Branch, National Institute of Allergy and Infectious Diseases, Bethesda, Maryland, United States of America; 4 Infectious and Inflammatory Disease Center, Sanford-Burnham Medical Research Institute, La Jolla, California, United States of America; 5 Seattle Biomedical Research Institute, Seattle, Washington, United States of America; Icahn School of Medicine at Mount Sinai, United States of America

## Abstract

Recent studies have shown high usage of the *IGHV1-69* germline immunoglobulin gene for influenza hemagglutinin stem-directed broadly-neutralizing antibodies (HV1-69-sBnAbs). Here we show that a major structural solution for these HV1-69-sBnAbs is achieved through a critical triad comprising two CDR-H2 loop anchor residues (a hydrophobic residue at position 53 (Ile or Met) and Phe54), and CDR-H3-Tyr at positions 98±1; together with distinctive V-segment CDR amino acid substitutions that occur in positions sparse in AID/polymerase-η recognition motifs. A semi-synthetic *IGHV1-69* phage-display library screen designed to investigate AID/polη restrictions resulted in the isolation of HV1-69-sBnAbs that featured a distinctive Ile52Ser mutation in the CDR-H2 loop, a universal CDR-H3 Tyr at position 98 or 99, and required as little as two additional substitutions for heterosubtypic neutralizing activity. The functional importance of the Ile52Ser mutation was confirmed by mutagenesis and by BCR studies. Structural modeling suggests that substitution of a small amino acid at position 52 (or 52a) facilitates the insertion of CDR-H2 Phe54 and CDR-H3-Tyr into adjacent pockets on the stem. These results support the concept that activation and expansion of a defined subset of *IGHV1-69*-encoded B cells to produce potent HV1-69-sBnAbs does not necessarily require a heavily diversified V-segment acquired through recycling/reentry into the germinal center; rather, the incorporation of distinctive amino acid substitutions by Phase 2 long-patch error-prone repair of AID-induced mutations or by random non-AID SHM events may be sufficient. We propose that these routes of B cell maturation should be further investigated and exploited as a pathway for HV1-69-sBnAb elicitation by vaccination.

## Author Summary

The quest for universal influenza vaccine has gained wide interest with the discovery of human neutralizing antibodies that are able to variably cross neutralize and protect against different influenza strains, subtypes, groups and lineages. These antibodies, which bind to a highly conserved epitope in the hemagglutinin stem, are often encoded by rearranged *IGHV1-69* germline genes that alone make contact with HA and prevent virus entry and emergence of escape mutants. Our study was undertaken to gain an understanding of what structural requirements enable a rearranged *IGHV1-69* Ab to become a potent cross-neutralizing antibody. We found that in addition to a critical amino acid triad consisting of a pair of anchor residues in CDR-H2 and a properly positioned CDR-H3 Tyr, distinctive V-segment substitutions that arise in positions that are distinct from phase I AID somatic hypermutation (SHM) hotspot motifs are often required. As few as two V-segment SHM can fulfill this role which appears to facilitate the optimal binding of CDR-H2 Phe54 and CHR-H3-Tyr into adjacent hydrophobic pockets in the HA stem. These studies provide new information on the SHM requirements for *IGHV1-69*-encoded B cells to produce HV1-69-sBnAbs and suggest that there may exist alternative routes to their elicitation by vaccination.

## Introduction

Vaccination remains the principle means of preventing seasonal and pandemic influenza and its complications. A “universal” influenza vaccine that induces broad immunity against multiple subtypes of influenza viruses has been a long sought goal in medical research. The discoveries of human broadly neutralizing “heterosubtypic” antibodies binding to various epitopes on the conserved stem domain of HA [1]–[9] have reignited efforts to develop such a vaccine. These sBnAbs were identified either by panning phage-Ab libraries [1]–[3], [7], or were recovered from B-cells of infected and vaccinated influenza donors [8], [10]–[13]. However, only very low concentrations of sBnAbs are detected in the sera of seasonal influenza [10] or H5N1 vaccinees, or in commercial intravenous immunoglobulin (***IVIG***) preparations [14]; with notable exceptions being in the response to pdm2009 H1N1 strains and the 1976 swine-flu H1 vaccine [15], [16].

sBnAbs are shown to originate from various heavy chain V-segment germline genes. Interestingly, many of these studies (**Table S1 in Text S1**) have reported on the high utilization of the *IGHV1-69* germline gene in broadly neutralizing antibodies against the stem domain of group 1 influenza A viruses (HV1-69 sBnAbs). While *IGVH1-69* germline gene is highly utilized in the population [17], the regulation of this germline gene usage during development and adaptive immune responses has only recently been reexplored [18] following some initial investigations [19]–[21]. In addition, details of the molecular events that are involved in the elicitation of HV1-69-sBnAbs by vaccination or seasonal influenza infection remains unknown. The highly immunogenic globular head [1], [10], [11] is thought to be a main impediment for sBnAb elicitation as the stem epitopes have been shown to be readily accessible to sBnAbs [22].

In this study we sought to better define the V-segment amino acid substitutions and CDR-H3 amino acids within rearranged *IGHV1-69* germline genes that are preferentially used to allow an *IGHV1-69* germline based Ab to become a potent HV1-69-sBnAb. Analysis of 38 HV1-69-sBnAbs recovered from 8 laboratories (**Table S1 in Text S1**) indicates that broad-spectrum binding and neutralization is conveyed by a triad of critical anchor residues composed of two CDR-H2 residues including a hydrophobic residue at position 53 and Phe54, and properly positioned CDR-H3 tyrosines. In addition, we define distinctive V-segment mutations within the CDR H1/H2/H4 loops. Moreover, these V-segment mutations occur in positions that are sparse in activation-induced cytidine deaminase (AID) and polymerase eta (pol η) consensus “hot-spot” motifs. Together with panning of a semi-synthetic *IGHV1-69* Ab library against H5/H1 HAs, mutagenesis studies, and structural modeling we demonstrate that HV1-69-sBnAbs can be evolved from a *IGHV1-69* germline gene with as few as two V-segment substitutions with one occurring at CDR-H2 Ile52Ser, and by properly positioned Tyr in the CDR-H3 domain. The CDR-H2 substitutions at positions Ile52Ser and Pro52aGly/Ala are predicted to function not by making new contacts with the epitope themselves, but rather by enabling conformational changes within the CDR loops that facilitate optimal insertion of two major anchor residues CDR-H2 Phe54 and CDR-H3-Tyr98 into adjacent pockets in the stem. Our immunogenetic and structural studies demonstrate that the generation of critical SHM for HV1-69-sBnAbs does not occur through the classical phase I AID repair mechanism that takes place directly under WRCY/RGYW motifs, instead by phase 2 long-patch error-prone repair or random non-AID SHM events. Further, these results suggest that the secondary AID repair mechanisms as described here may not require B cell recycling/reentry in the germinal center [23], rather by alternative routes such as short term entry into the germinal center or in a specialized extra-follicular location such as the marginal zone [24].

## Results

### HV1-69-sBnAb CDR-H3 Tyrs together with CDR-H2 Phe54 mediate high affinity binding to adjacent pockets in the HA stem

**Figure 1:**
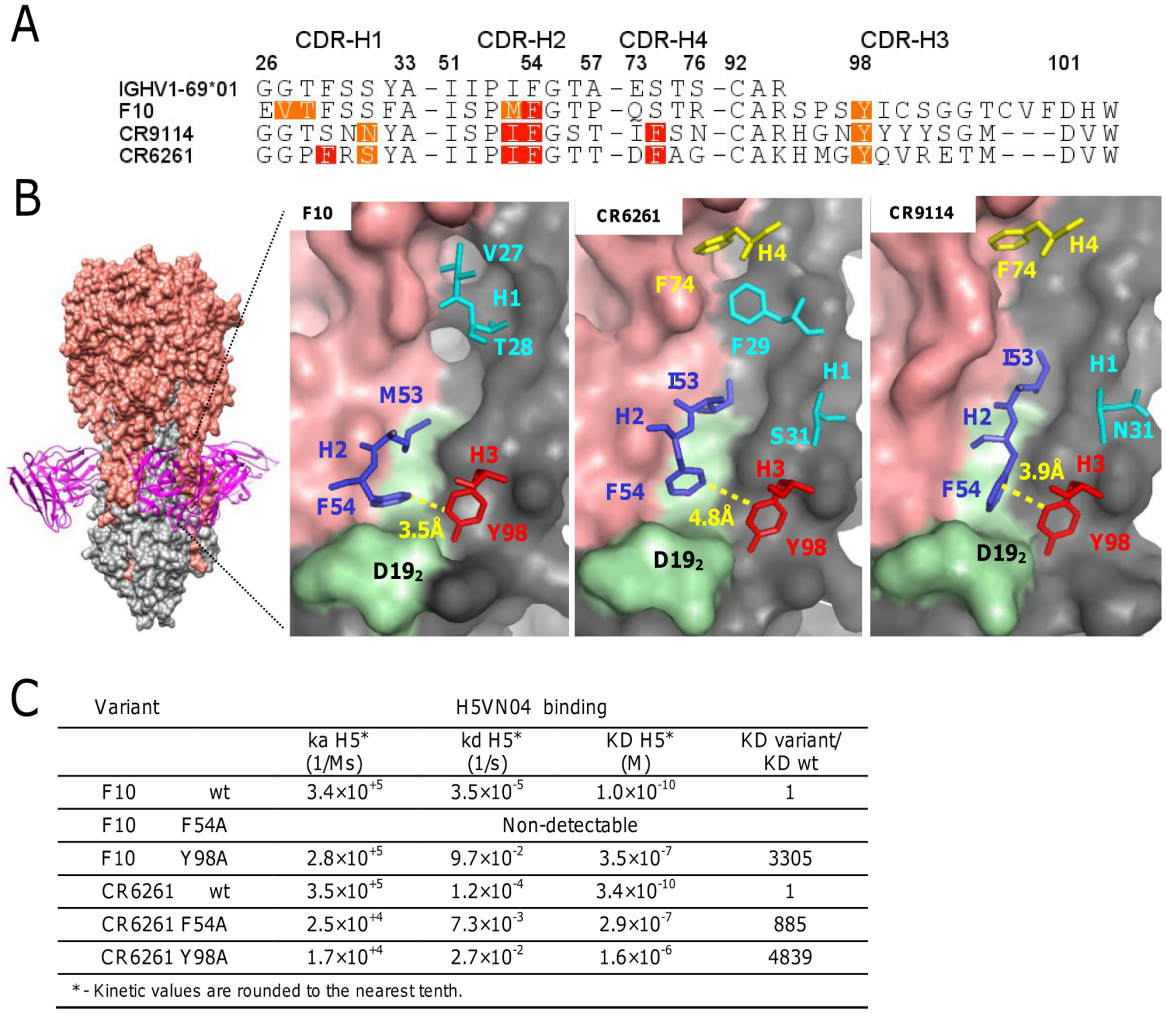
The structural basis of HV1-69-sBnAb similarity. **A**) The ANCHOR web server [25] was used to identify heavy chain CDR residues that make favorable contacts (−1 kcal/mol >−3 kcal/mol orange) and highly favorable binding contacts (<−3 kcal/mol red) in the co-crystal structures of F10 (PDB: 3FKU) CR6261 (PDB: 3GBM), and CR9114 (PDB: 4FQI). **B**) Left - The location of F10 binding on the HA is shown with HA1 colored in salmon and HA2 colored in grey. Right panels – The location of the CDR residues identified in **A**). In light green is the HA2 fusion peptide from Trp21_2_-to-Val18_2_. **C**) Binding kinetics data of F10, CR6261, and the respective variants of F10 F54A, F10 Y98A, CR6261 F54A and CR6261 Y98A, against H5VN04.

The co-crystal structures of the HV1-69-sBnAbs, F10 [1], CR6261 [4], and CR9114 [7] with H5VN04 established that binding is mediated exclusively by the *IGHV1-69* heavy chains. To further explore binding similarities in the co-crystal structures, binding free energy contributions for the heavy chain CDR residues were estimated by using the ANCHOR server [25] (**Figure 1A**). This analysis revealed the under recognized importance of CDR-H3-Tyr98 as another common anchor residue to the already two known hydrophobic residues at position 53 (Ile/Met), and the conserved Phe at position 54 [1], [4], [26]. In these three HV1-69-sBnAb structures Tyr98 is located in close proximity to Phe54 (∼4 Å) and is the only contact residue in which the side chain adopts a single conformation in its binding pocket (**Figure 1B**), and forms a strong H-bond with the fusion peptide (the main chain carbonyl of Asp19_2_). To further validate the importance of CDR-H3 Tyr98, the binding kinetics of F10 and CR6261 Y98A variants to H5VN04 were compared to wt and F54A variants (**Figure 1C**). Binding kinetic studies showed that Y98A reduced binding by >3000-fold and >4,000-fold for F10 and CR6261, respectively. In addition, the F54A substitution completely ablated binding of F10 and reduced the CR6261 binding by >800-fold, which is consistent with previous studies [3], [26]. Thus, the poor binding kinetics of the Y98A variants further substantiates CDR-H3 Y98 as a major contributor to HA binding.

### Identifying distinctive CDR-H3 and V-segment molecular signatures in HV1-69-sBnAbs

**Figure 2:**
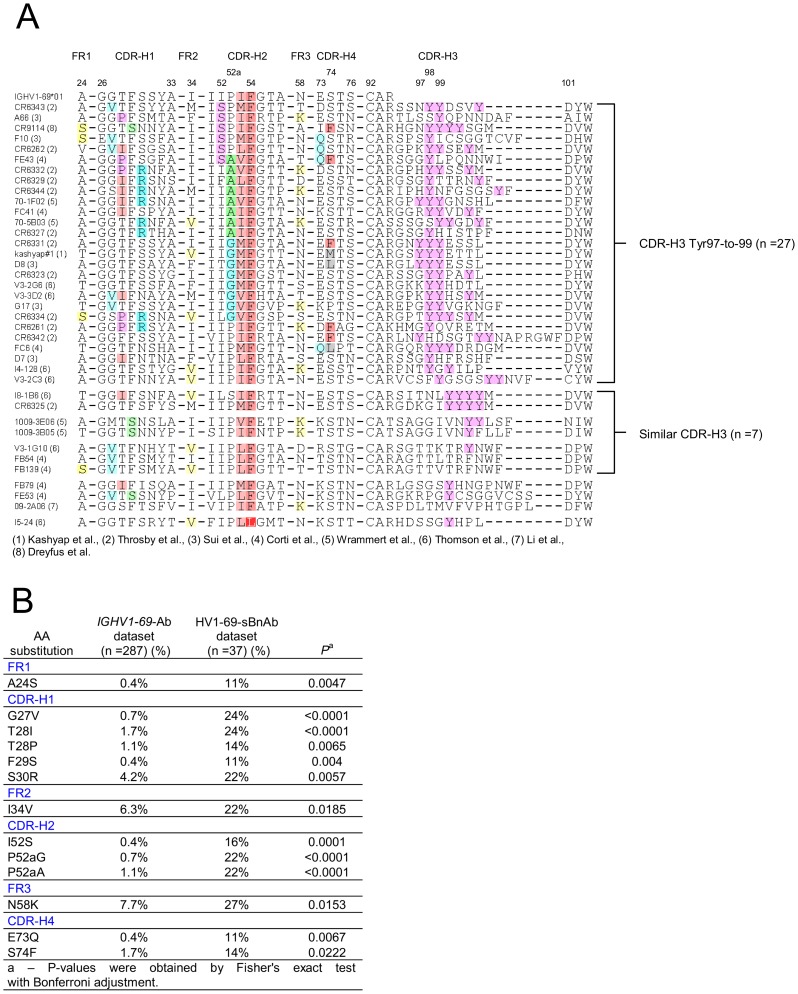
Characterization of HV1-69-sBnAbs VH domain. **A**) Alignment of 38 published HV1-69-sBnAbs is shown with highlights referring to hydrophobic residues at position 53 (light plum), the conserved Phe54 (dark plum), the occurrence of CDR-H3-Tyr (pink) residues. Other highlights refer to panel **B**), which describes the result of a Fisher's exact test with Bonferroni adjustment that compared V-segment amino acid substitutions diversity and frequency of the 37 51p1 allele related HV1-69-sBnAbs with that of a reference *IGHV1-69* 51p1 allele related Ab dataset. 13 amino acid substitutions were determined to uniquely associate with the HV1-69-sBnAb dataset (P<0.05).

The conserved triad of a hydrophobic residue at position 53, Phe54, and Tyr98, led us to explore the commonality of these residues in published HV1-69 sBnAbs (**Table S1 in Text S1**). **Figure 2A** shows that hydrophobic residues are always found at CDR-H2 position 53, Phe54 is nearly invariant, and CDR-H3 tyrosines are found in 35/38 HV1-69 sBnAbs. Further immunogenetic analysis demonstrated that 37 of these HV1-69 sBnAbs belong to the *IGHV1-69* 51p1 allele group, all of which encode the critical Phe54 (**Figure S1A–B in Text S1**) whereas the D-segments or J-segments are highly diverse (**Figure S1C–D in Text S1**).

To identify possible distinctive V-segment amino acid substitutions in the HV1-69-sBnAb Ab dataset, we compared all of their amino acid substitutions with a reference V-segment dataset composed of functional, non-duplicated *IGHV1-69*-51p1 allele based Abs (IgBlast, n = 287). Thirteen distinctive HV1-69-sBnAb V-segment substitutions were identified using Fisher's exact test with Bonferroni adjustment (highlighted in **Figure 2A** and respective frequencies shown in **Figure 2B**). When the HV1-69-sBnAbs are ordered according to the occurrence of the distinctive CDR-H2 substitutions, a prominent cluster of Ser52, Gly52a, and Ala52a mutations is seen in 21/27 antibodies with CDR-H3 domains that are also characterized by tyrosines in positions 97-to-99 (**Figure 2A**). The Gly52a substitutions are associated with dual or triple CDR-H3, and Ala52a substitutions are strongly associated with the distinctive substitution of Arg30 (6/8). Furthermore, distinctive CDR-H4 (**Figure S2 in Text S1**) substitutions of Gln73 and Phe74, and CDR-H1 Pro28 and Arg30 only occur with HV1-69-sBnAbs containing CDR-H3-Tyr97-99. Of the other 10 HV1-69-sBnAbs not characterized by CDR-H3-Tyr97-to-99, several high CDR-H3 sequence similarities were observed, which allowed grouping of 7 of these HV1-69-sBnAbs into three sub-clusters.

### Studying the origins of the distinctive HV1-69-sBnAb V-segment CDR substitutions

**Figure 3:**
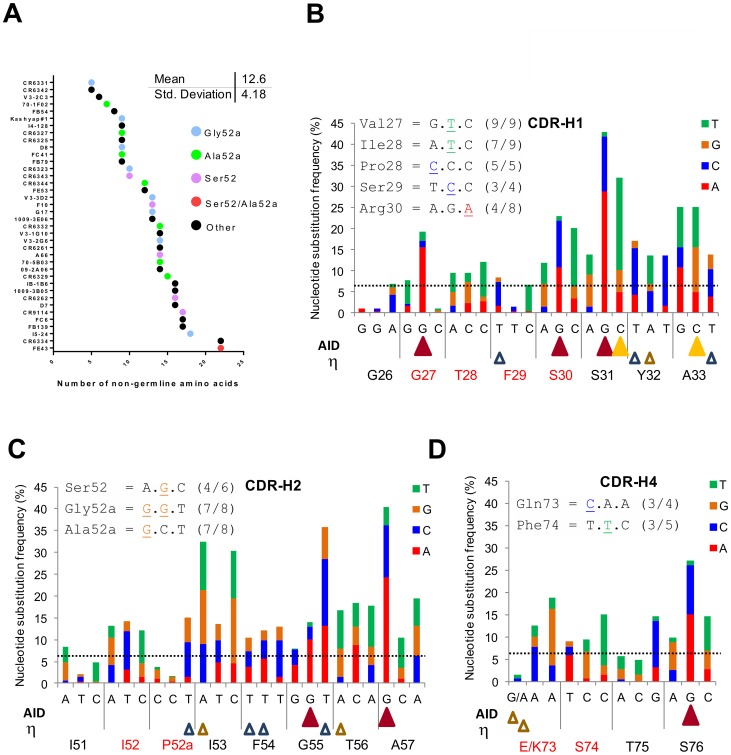
Studying the location of SHM hotspots and nucleotide substitutions frequencies in the *IGHV1-69* reference Ab dataset. **A**) Number of V-segment substitutions observed in 38 HV1-69-sBnAbs with color notations that designate the HV1-69-sBnAbs characterized by the distinctive CDR-H2 Ser52 or Ala/Gly52a and “other” which do not contain CDR-H2 Ser52 or Ala/Gly52a. (**B–D**) **Upper panel** – the common nucleotide substitutions that generated the distinctive amino acid substitutions in the respective CDR domain. **Main panel** - The *IGHV1-69* 51p1 Ab reference dataset was studied for substitution frequency of nucleotides in the V-segment CDRs and for location of AID and polη hotspots (AID = WRCY yellow solid triangle/RGYW dark red solid triangle, Polη WA brown open triangle/TW blue empty triangle. The horizontal line shows the mean±SD (6.44±7.23) of non-germline nucleotide substitution frequency observed for FR regions to serve as a reference.

**Figure 3A** shows that HV1-69-sBnAbs are characterized by a mean of 12.6±4.2 V-segment substitutions that ranges in a continuum from 5 in CR6331/CR6432 to 22 in FE43/CR6334. This is in marked distinction to the much higher number of somatic mutations that are found in anti-HIV gp120 CD4-binding-site sBnAbs that show biased use of the *IGHV1-02* germline gene [27]. Furthermore, the distinctive substitutions are distributed across this continuum as exemplified by the CDR-H2 Ser52 and Gly/Ala52a substitutions.

The nucleotide changes in CDR H1, H2 and H4 that are responsible for these distinct HV1-69 sBnAb substitutions are shown to occur mostly by one nucleotide and not multiple nucleotide changes in the codon (**Figure 3B-D** **upper panel inserts**). This observation led us to further explore the nucleotide substitution frequencies in the same non-duplicated *IGHV1-69* reference dataset and its relation to the location of AID and pol η “hotspot” somatic hypermutation (SHM) motifs [28], [29]. In agreement with the study by Clark et al [30], positions of high nucleotide substitution frequency are mostly found directly under AID hotspot motifs. It is also noticeable that AID motifs are found to concentrate towards the 3′ end of the CDR loops. In CDR-H1 4/5 AID hotspots are located 3′ to position 29, in CDR-H2 2/2 are located 3′ to position 54 and in CDR-H4 1/1 is located 3′ to position 75. Notably, of 10 distinctive substitutions on CDR-H1,H2 and H4 only two, Val27 and Arg30 are directly under AID motifs. The Gly27Val substitution has the unusual property of occurring by dG-to-dT transversion instead of the phase Ia AID associated dG-to-dA transition to Gly27Asp, suggesting it occurred by a phase Ib short patch base excision repair (BER) mechanism and/or by long patch (BER)/mismatch repair (MMR) phase 2 mechanism [28]. The polη (open triangles) SHM motifs are more disperse, however the 5′ end of both CDR-H1/H2 domains is also sparse of this SHM motif. Hence, it appears that in response to influenza infection or vaccination mutations in the V region are not the result of the direct biochemical action of AID, but rather depend on the error-prone BER and MMR of the AID-induced mutation.

### Exploring the importance of distinctive HV1-69-sBnAbs substitutions through use of a semi-synthetic *IGHV1-69* library that bypasses AID/Pol η restrictions

In consideration of the immunogenetic DNA repair mechanisms that may be responsible for the critical CDR-H1/H2/H4 positions in HV1-69 sBnAbs noted above, we generated a semi-synthetic phage-antibody library (5 displayed Abs/phage) to bypass restrictions that may be related to the SHM machinery. The library was designed with a low V-segment amino acid substitution frequency (1.9±1.1) and incorporated 9 of the 13 distinctive HV1-69 sBnAb amino acid substitutions at a frequency no higher than 10% and with a completely randomized CDR-H3 of varying length (**Supporting text in Text S1**). The library is strongly skewed towards selection of Ab-members that display germline residues. For example, the combination of the V-segment germline residues of CDR-H1 Gly27 (90%) and CDR-H2 Ile52 (71%) with CDR-H3 Tyr98 (11%) is expected to occur in 7% of the phage members whereas the combination of the distinctive HV1-69-sBnAb substitutions of Val27 (10%), Ser52 (10%) with CDR-H3 Tyr98 is (11%) expected to occur in 0.11% of the phage members (**Figure S3 in Text S1**).

**Figure 4:**
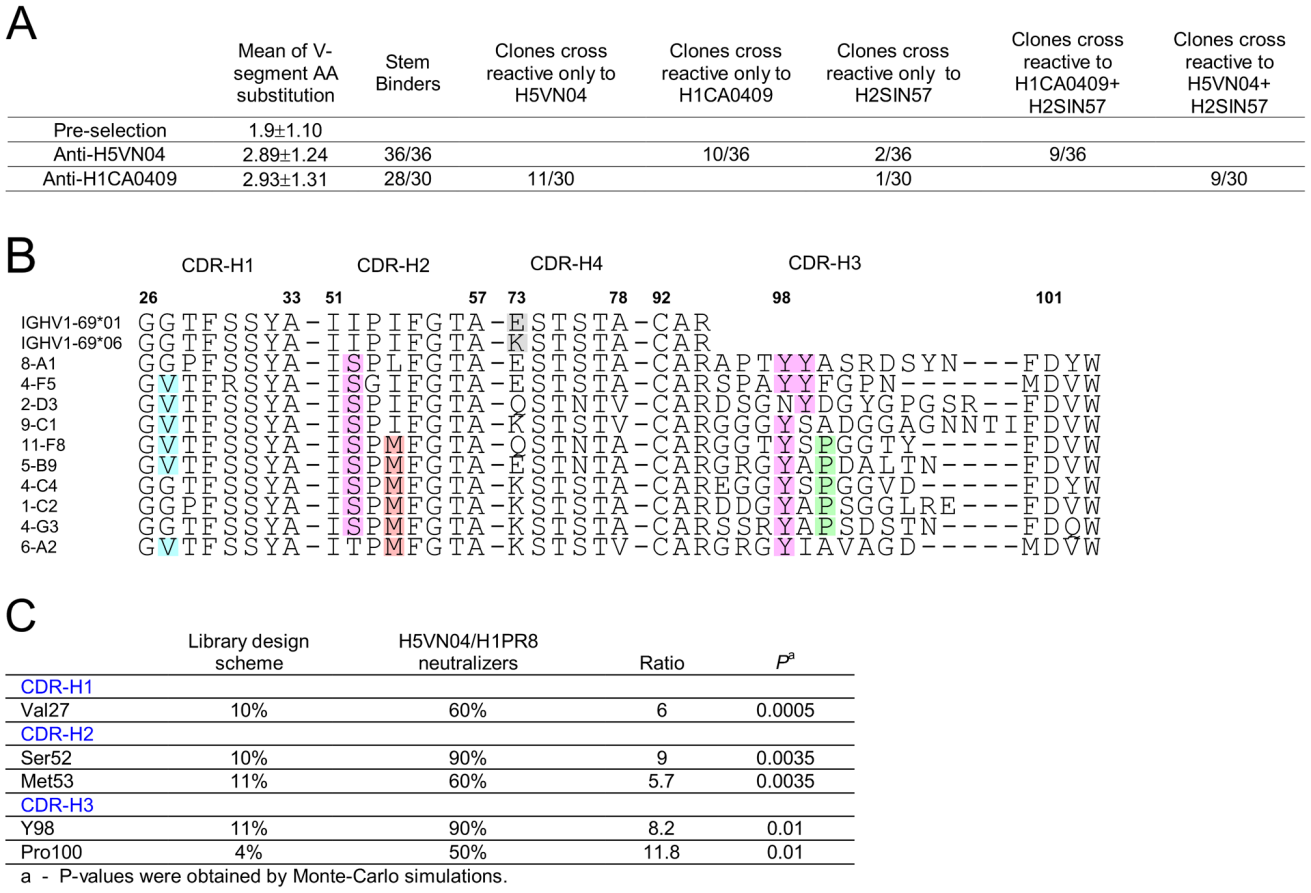
Semi-synthetic HV1-69 phage-Ab library yields potent anti-H5VN04/H1CA0409 Abs characterized by a minimal V-segment amino acid substitutions. **A**) Characterization of binding activities of anti-H5VN04 and anti-H1CA0409 phage-Abs isolated from the semi-synthetic HV1-69 phage-display library. Sequences are detailed in **Figure S4 in Text S1**. **B**) Heavy chain CDR sequences of anti-H5VN04 phage Abs characterized by >95% neutralization activity against both H5VN04 and H1PR8 pseudotyped viruses. The 5 highlighted residues in the CDRs refer to panel **C**) which describes the result of a Chi square statistical analysis approach used to identify residues that were significantly enriched as compared to their frequency in the library (P<0.05). Also highlighted are Tyr99 and position73 in the *IGHV1-69* germline sequences.

Panning the library against the H5-VN04 or H1CA0409 trimeric HA proteins resulted in the isolation of 36 and 30 unique phage-Ab clones, characterized by low V-segment amino acid substitution frequency of 2.89±1.24 and 2.93±1.31, respectively (**Figure 4A**). F10 competition assays performed with H1CA0409, H2SIN57 or H5VN04 coated on MSD plates further indicated that all anti-H5VN04 phage Ab are stem directed and 10 cross-reacted with H1CA0409; 2 cross-reacted with H2 A/Singapore/1/1957 (H2SIN57); and 9 cross-reacted with both H1CA0409 and H2SIN57 (**Figure S4A in Text S1**). Likewise, 28/30 anti-H1CA0409 phage Abs target the stem, and 11 cross-reacted only with H5VN04; 1 cross-reacted only with H2SIN57; and 9 cross-reacted against both H1CA0409 and H2SIN57 (**Figure S4D in Text S1**). Of the two non-stem binder only one cross-reacted against H5VN04.

Heterosubtypic neutralization activity was tested for thirty-one anti-H5VN04-stem phage-Abs by using H5VN04 and H1PR8 pseudotyped viruses. Ten of these phage-Ab characterized by >95% neutralization activity against both strains are shown in **Figure 4B**. Chi^2^ based statistical analysis of these 10 sBnAbs that compares the frequency of substituted amino acids before and after selection (**Figure 4C**) revealed a sequence solution conveyed by two distinctive HV1-69-sBnAb substitutions CDR-H1 Val27 (6/10) and CDR-H2 Ser52 (9/10) together with CDR-H3 CDR-H3-Tyr 98 (9/10). Overlapping non-HV1-69-sBnAb specific substitutions of Met53 (6/10) and CDR-H3 Pro100 (5/10) were also found in this pool. In the remaining pool of anti-H5VN04 phage-Abs (**Figure S4B in Text S1**), the substitutions of CDR-H4 Asn76 and CDR-H3 Gly97, Tyr99, and Gly100B were also significantly enriched.

**Figure S4A in Text S1** also shows that the dominant sequence motif of Ser52/Tyr98 occurs in both the heterosubtypic (16/21) and the non-heterosubtypic anti-H5VN04 phage-Ab subsets (8/15). In order to understand if heterosubtypic activity of phage-Abs characterized by the Ser52/Tyr98 sequence motif is associated with other amino acids, the composition of the CDR-H3 domain of the two subsets was analyzed separately for the occurrence of enriched residues. The statistical analysis in **Figure S4C in Text S1**shows that in the heterosubtypic Ser52/Tyr98 subset Tyr99, Pro100, and Gly100B were significantly enriched whereas no significant enrichment of these same residues were found to occur in the CDR-H3 of the non-heterosubtypic subset. Furthermore, although not statistically significant, the high frequency of glycines in the heterosubtypic subset is also shown in **Figure S4A in Text S1** to occur 5′ to CDR-H3-Tyr98 where 46% of the amino acids at positions 95-to-97 are glycines as opposed to the non-heterosubtypic subset where 21% are glycines at these positions. This analysis suggests that a flexible CDR-H3 loop is beneficial in mediating heterosubtypic activity for anti-H5VN04 stem binders characterized by CDR-H2 Ser52 and CDR-H3-Tyr98.

A similar amino acid enrichment profile was also observed in the H1CA0490 phage-Ab pool (**Figure S4D–E in Text S1**) that are characterized by the dominant pair of Ser52/Tyr98 as well as by CDR-H1 Val27, CDR-H4 Asn76 and CDR-H3 Gly97. These substitutions are also shown to dominate the heterosubtypic phage-Ab subset whereas in the non-heterosubtypic subset CDR-H1 Val27 and CDR-H2 Ser52 appear only once (1/9) and Tyr residues in positions 98 or 99 appear only in three phage-Abs (3/9).

The predominant recovery of Ser52 over Gly52a and Ala52a encoding phage-Abs from the panning campaigns despite similar coding frequency in the library (**Figure S3 in Text S1**) was unexpected but might be explained in view of the fact that the Gly52a subset is restricted to double or triple tyrosines in the CDR-H3 domain, whereas the Ala52a subset is shown to be strongly associated with CDR-H1 Arg30 (6/8) (**Figure 2A**). In contrast, the Ser52 does not appear to be as strongly associated with other V-segment substitutions. Thus, the incorporation of Ser52 likely provides a higher diversity of structural solutions than that of Gly52a and Ala52a as follows: the frequency of phage-Ab members characterized by CDR-H2Ser52/CDR-H3-Tyr98 is expected to be 1%, the frequency of phage members characterized by CDR-H2 Gly52a/CDR-H3-Tyr98 and 99 is expected to be ∼0.14% and the frequency of phage members characterized by CDR-H1 Arg30/CDR-H2 Ala52a and CDR-H3-Tyr98 is ∼0. 11%.

### Confirming the importance of the CDR-H2 substitutions in HV1-69-sBnAbs

**Figure 5:**
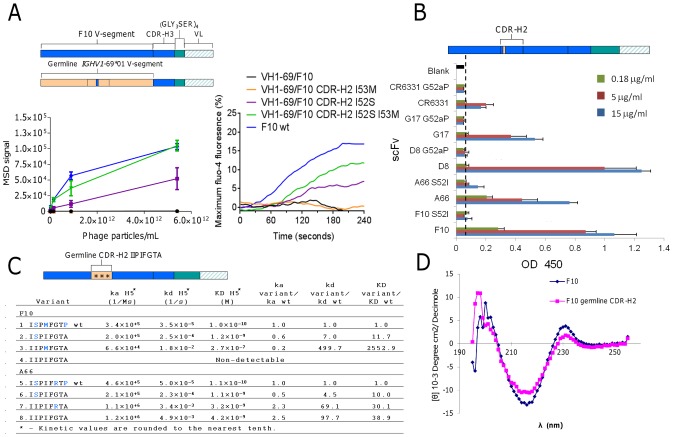
Validating the structural role of Ser52 in HV1-69-sBnAbs. **A**) F10 V-segment germline variants were analyzed for H5VN04 binding in the phage-Ab (5 scFv/phage) format by MSD ELISA *(left)* and for their ability to activate B-cell when expressed as B-cell receptors in the presence of H5VN04 *(right)*. **B**) HV1-69-sBnAb variants of S52I in F10 and A66, G52aP in CR6331, G17 and D8 were analyzed for H5VN04 reactivity by ELISA. **C**) Kinetic analysis by Biacore of F10 and A66 CDR-H2 variants against purified H5VN04. Residues colored in blue are non-germline amino acids. **D**) Circular dichroism measurement of F10 and the non-H5 reactive variant characterized by a germline configured CDR-H2 shows a highly similar CD profile for both constructs.

Since Ser52 was the dominant sequence solution obtained from the panning campaigns of the synthetic library we next sought to test the relative importance of Ser52 in F10 when the V-segment was converted to the non-mutated IGHV1-69*01 form (VH1-69/F10) and then back introduced with Ser52 and Met53 separately and together. In order to utilize avidity to increase detection of weak interactions, the VH1-69/F10 variants were either expressed on the surface of phage particles and binding tested with an MSD ELISA assay (**Figure 5A**, *left*); or expressed as B-cell receptors and analyzed for their ability to activate B-cells through cross-linking with purified H5VN04 trimeric HA (**Figure 5A**, *right*). In both cases, we detected no binding of H5VN04 with either the germline or the I53M variant. However, the I52S variant was active, and the I52S/I53M variant had even higher activity.

To further validate the structural role of the distinctive CDR-H2 substitutions in the context of the fully mutated HV1-69-sBnAbs we mutated five representative HV1-69-sBnAbs carrying either I52S or P52aG mutation back to the germline gene residue. All five variants had drastically reduced or abolished binding reactivity to H5VN04 (**Figure 5B**). In comparison, a revertant proline substitution at CDR-H2 position 57 in F10 and A66 resulted in enhanced or no change in H5 binding, respectively (data not shown). We also constructed F10 and A66 CDR-H2 germline variants then back introduced the substitutions of Ser52 (F10/A66), Met53 in F10, and Arg55 in A66 (**Figure 5C**). The kinetic data shows only small differences in association rate (k_a_) constants among the wild-type and variants, whereas much greater effects were observed with the dissociation rate (k_d_) constants. Replacing wild-type CDR-H2 with the germline sequence (IIPIFGTA) led to a 98-fold higher dissociation rate in A66 (construct #5 vs. #8) and no detectable binding activity in F10 (construct #1 vs. #4). Circular dichroism (CD) analysis indicated that protein misfolding is not responsible for loss of binding as a similar profile was observed for the F10 germline CDR-H2 variant and F10 wt (**Figure 5D**). Restoring the single amino acid of Ser52 (ISPIFGTA) (mutants #2 & 6) resulted in recovery of binding kinetics for both sBnAbs, as seen by the dramatic improvement in k_d_ values. Other single amino acid CDR-H2 reversions (F10 IIPMFGTA, A66 IIPIFRTA) (mutants #3 and 7) did not restore binding to the same extent and showed extremely fast k_d_ rates (**Figure 5C**).

### Understanding the structural role of the HV1-69-sBnAb distinctive CDR-H2 substitutions

**Figure 6:**
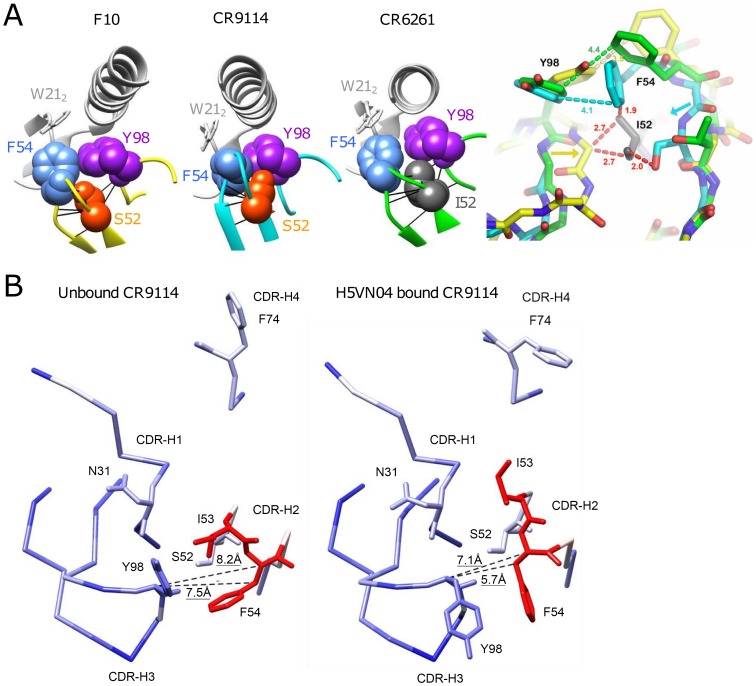
Understanding the structural role of the distinctive CDR-H2 amino acid substitutions in HV1-69-sBnAbs. A) VDW contact analysis (black lines) shows that Ser52 of F10 and CR9114 (orange), and Ile52 of CR6261(gray) make only intramolecular contacts; i.e., do not form contacts with their respective H5VN04s. Antibodies are shown in color; HA is in light gray. At far right, steric consequences of the germline Ile52 and the Ile52Ser substitutions are shown when the Abs are overlaid on their framework residues (RMSD ∼0.5 Å). Comparing structures of the HV1-69-sBnAbs, centered on Ile52 of CR6261 (green), with F10 (yellow) and CR9114 (cyan), the Ile52Ser mutation in F10 and CR9114 enables the 2 strands to come closer together, as indicated by the yellow and cyan arrows. Distances in red indicate hypothetical steric clashes (<3 Å) that would be created if Ile52 were present in CR9114 and F10. B) Comparison between the unbound (PDB 4FQH, left) and H5VN04-bound structures (PDB 4FQI, right) of CR9114, colored according to the magnitude of structural change after superposition on the main-chain of the VH domain (from blue = 0 Å, through white = 1 Å, to red = 1.8 Å). CDRs and side-chains of the major contact residues are shown, as depicted in Figure 1A. Distances between the Cα and Cβ atoms of Phe54 and the Cα atom of CDR-H3 Tyr98 (shown as dashed lines) are indicated. Large rotations of the side chains of CDR-H3 Tyr98, CDR-H2 Phe54 and CDR-H2 Ile53 are also evident, as previously noted [7].

Ser52 of F10 and CR9114 do not form high energy contacts with the respective H5VN04 HAs, as evidenced by van der Waals (VDW) contact analysis (**Figure 6A**); thus, direct contacts with antigen are not the source of the dramatic effect of the Ile52Ser mutation. Analyzing the contacts between Phe54 and Tyr98 in the H5VN04-bound structures shows Phe54 making close (∼3.7 Å) orthogonal contacts with Tyr98 in the F10 and CR9114 complexes, whereas for CR6261, the 2 rings are further apart (4.8 Å) and nearly coplanar. *In silico* mutagenesis of Ser52 to germline Ile52 in a F10 model in which its amino acid side chains are allowed to rotate, shows an increase in number of VDW contacts (**Figure S5 in Text S1**). Furthermore, it was noted that Ile52 could not be accommodated in the F10 and CR9114 H5-bound structures in which the amino acids side chains were fixed. This can be visualized by structural alignment of the CDR-H2 loops of F10, CR9114 and CR6261, which shows Ile52 sterically clashing with both F10 and CR9114 (**Figure 6A** **, right panel**). In addition, structural alignment between unbound CR9114 and the H5-bound structure (**Figure 6B**) shows that only CDR-H2 residues, Ile53 and Phe54, adopt markedly different positions (>1.7 Å shifts) following HA binding. Distance analysis suggests an induced-fit process, in which the Cα-Cβ atoms of Phe54 are shifted closer to the CDR-H3 Tyr98 domains in the bound state. We propose that this induced fit process could not occur if the large germline Ile52 is maintained in that position.

The close distance between Phe54 and CDR-H3-Tyr is also expected to be a structural feature in HV1-69-sBnAbs carrying the P52aG/A substitutions, since, like I52S, they are characterized by a narrow distribution of CDR-H3 Tyr residues clustered around positions 97-to-99 (**Figure 2A**). *In silico* mutagenesis suggests that replacing germline Pro52a in the non-mutated *IGHV1-69* germline-based Ab 1-69/B3 [31] with Gly52a also leads to a reduction in VDW contacts (**Figure S5 in Text S1**).

## Discussion

Development of a “universal” influenza vaccine that is aimed at the elicitation sBnAbs should remain a high priority for global health and national biosecurity. Such a vaccine should preferably induce sBnAbs of high diversity that target various stem epitopes and would not allow generation of escape variants. In this respect the *IGHV1-69* germline gene based sBnAbs have the property of showing recalcitrance to neutralization escape [1], [3], [10]. This feature and the isolation of HV1-69-sBnAbs from various donors led us to further characterize reported HV1-69-sBnAbs and understand what are the requirements for *IGHV1-69* germline based Abs to become a potent HV1-69-sBnAb. Starting with a comparative structural analysis of three published HV1-69-sBnAbs, our analysis shows that a shared CDR-H3-Tyr98 assumes identical conformations within its own stem pocket and forms a strong H-bond with the fusion peptide (**Figure 1A–B**). Functional importance of Tyr98 was shown by mutagenesis studies and by their high frequency of recovery from panning studies and is also implied from the occurrence of the CDR-H3-Tyr 97–99 cluster in 71% of the reported HV1-69-sBnAbs (**Figure 2A**). Hence, this analysis adds a third common anchor residue to the two well described contact amino acids of hydrophobic Met/Ile53 and Phe54, and provides a new view on the contribution of the CDR-H3 loop on HA binding [4], [32].

Immunogenetic analysis shows that HV1-69-sBnAbs CDR-H3 Tyrs arise from a diverse repertoire of D-segments, whereas 37/38 of the HV1-69-sBnAbs are shown to utilize *IGHV1-69* V-segment alleles (6/13) characterized by CDR-H2 Phe54 (i.e., the 51p1-allele group). This allelic bias cannot be attributed to the frequency of 51p1 and hv1263 alleles in the population [33] and further supports a critical role of Phe54 (**Figure S1 in Text S1**). These results suggest that precursor pool of HV1-69-sBnAbs preferentially requires the triad of anchor residues of CDR-H2 Ile53/Met53, CDR-H2 Phe54 and CDR-H3-Tyrs. Based on these immunogenetic findings, it appears that HV1-69-sBnAb elicitation may be constrained when an individual is 51p1-null and expresses only Leu54 from the hv1263 alleles (circa 20% of the population) [33] (Avnir et al in preparation). We observed a low frequency (circa 6–7%) of Leu54Phe substitutions in the hv1263 allelic group by NGS sequencing of our 27 billion member non-immune Ab-phage library (data not shown).

Sequence similarities among the HV1-69-sBnAbs were also observed in the V-segments. Our statistical analysis identified thirteen amino acid substitutions that are distinctive to the HV1-69-sBnAb dataset when compared to *IGHV1-69* 51p1 germline based Abs obtained from the IgBlast database. Seven of these changes: CDR-H1 Pro28/Arg30, CDR-H2 Ser52/Gly52a/Ala52a, CDR-H4 Gln73/Phe74, were shown to occur in 24/27 (89%) HV1-69-sBnAbs that are characterized by CDR-H3-Tyr clustered at positions 97-to-99 (**Figure 2A**). Interestingly, six of the seven V-segment substitutions arise in CDR positions that are sparse in AID SHM motifs (WRCY/RGYW) (**Figure 3B–D**). Therefore, these mutations were generated by either error-prone long patch BER and MMR processes or by random SHM events. In addition, HV1-69-sBnAbs characterized by Gly52a are shown to associate with double or triple CDR-H3-Tyr and those characterized by Ala52a that are strongly associated with Arg30 and appear to represent unique molecular signatures for a subset of HV1-69-sBnAbs.

To further interrogate the distinctive HV1-69-sBnAb substitutions a semi-synthetic *IGHV1-69* library was designed aimed at bypassing SHM machinery restrictions, while maintaining low V-segment substitutions. H5/H1 panning campaigns resulted in the isolation of HV1-69-sBnAbs characterized by a low frequency of V-segment amino acid substitutions and a high frequency of CDR-H3-Tyr98/99 (**Figure S4 in Text S1**). The distinctive CDR-H2 Ser52 substitution was frequently recovered from both H5VN04 and H1CA0409 pannings (80.6% and 56.7%, respectively), and to a lesser extent (∼30%–40%) CDR-H1 Val27, CDR-H2 Met53, and Asn76 were also observed (**Figure S4 in Text S1**). The dominance of CDR-H2 Ser52 led us to investigate its functional importance. BCR and phage-Ab studies with *IGHV1-69/F10* reverted germline V-segment mutants showed the benefit of CDR-H2 Ser52 as a single mutation (**Figure 5A**) and mutagenesis studies confirm its importance in the natural HV1-69-sBnAbs (**Figure 5B–C**). Structural modeling (**Figure 6**) suggests that the CDR-H2 Ser52 and P52aG/A substitutions do not make direct contact with HA, rather they likely facilitate the ability of CDR-H2 Phe54 and CDR-H3 Tyr98 to come into close apposition and insert into adjacent pockets in the stem (**Figure 1B** **;** **Figure 6**). The findings of enriched glycine and proline residues opposed to CDR-H3-Tyr98/99, particularly for heterosubtypic Abs also adds support that CDR-H3 loop flexibility may facilitate the proper CDR-H2/H3 loop alignments for optimal binding. CR6261 lacks CDR2-H2 52/52a substitutions and uses a modified strategy which relies on distinctive substitutions occurring in the CDR-H1/H4 domain. The study of Lingwood et al [26] has shown that high H1 reactivity of CR6261 was restored only when 7 mutations, including T28P and S30R in CDR-H1 and heavily diversified CDR-H4 were back introduced into the *IGHV1-69* germline gene.

Current dogma suggests that robust B cell expansion to seasonal vaccination is often limited to B-cells that target more plentiful epitopes on the HA head domain that may outcompete for critical resources. Human immune response studies of the pdm2009 H1N1 virus have shown a relative shift in the antibody repertoire towards heterosubtypic Abs attributed to the highly divergent sequence of its head domain compared to seasonal H1N1 [11]–[13], [34]. Isolated mAbs from these studies are characterized by high frequency of SHM suggesting that these originated from preexisting small population of heterosubtypic memory B-cells that were expanded through increased interaction with immune cells including follicular T cells (Tfh) and their soluble mediators [12], [13], [35]–[37]. In contrast to the above, our results suggest that generation of HV1-69-sBnAbs may not require prolonged recycling/reentry of B cells into germinal centers to acquire additional SHMs since both the BCR studies (**Figure 5A**) and panning studies (**Figure S4 in Text S1**) demonstrated that as few as one (**Figure 5A**) or two (**Figure S4 in Text S1**) V-segment substitutions respectively, together with properly positioned Tyrs in the CDR-H3 domain can result in heterosubtypic binding and neutralization. This reasoning further suggests that HV1-69-sBnAb B-cells may be derived from a heterogeneous population of memory B cells, including B cells that exist the germinal center early without the need to extensively mutate their rearranged VH genes and IgM^+^CD27^+^ marginal zone (MZ) B cells that are proposed to develop outside of the germinal center [24]. B cell helper neutrophils (N_BH_) have been shown to trigger SHM in MZ B cells through an extrafollicular pathway that may not require T cells [38]. In support of this proposition the Abs reported by Throsby et al [3] (**Table S1 in Text S1**) are derived from MZ B cells and HV1-69-sBnAbs CR6331/CR6342 (**Figure 3A**) contain only 5 V-segment mutations and have critical substitutions that occur in non-classical SHM positions. In addition, it appears that generation of stem targeted mAbs does not require active germinal centers as the study of Keating et al [39] showed that mice treated with rapamycin and immunized with A/HK/x31 (H3N2) were able to generate Ab response that targets the stem domain of H5VN04 HA.

While there are reports of successful vaccination approaches that lead to robust elicitation of sBnAbs in animals [40]–[42], it remains to be determined if these approaches will elicit a diverse pool of sBnAbs in human [43]. We propose that, it is worthwhile to consider how to inclusively elicit HV1-69-sBnAbs in human vaccines because of their virologic potency. Why broad elicitation of naive B-cell derived HV1-69-sBnAbs was not the dominant component of the Ab response seen to pdm2009 H1N1 and how primary and recall sBnAb responses can be augmented by vaccination are questions yet to be answered. Similar to the evolving vaccination approaches that are being applied to HIV which include immunogen designs that target VRC01 precursor B-cells [44], selective clonal expansion of HV1-69-sBnAb precursor B-cells might be achieved by a stem-epitope containing immunogen [45]–[48] or by anti-idiotype [49] priming that results in stimulation of a larger *IGHV1-69* BCR precursor pool. In addition, the molecular signatures discovered in this study might be also useful for confirming success of such vaccination approaches in humans. For example, the Ab repertoire of individuals can be analyzed pre and post-influenza vaccination by using NGS [50]. In this regard, screening the antibodyome for the HV1-69-sBnAb molecular signatures as defined in this study would aid in quantitating their contribution to the sBnAb response. Likewise given the allelic variation of the Ig locus in the population, a complementary genomics approach should be considered to evaluate the role of *IGHV1-69* polymorphism in the sBnAb response [33], [51].

## Materials and Methods

### Materials

The anti-HA antibodies F10, A66, G17, and D8 were previously described in the study of Sui *et al* [1]. The mAbs CR6261 and CR6331 were synthesized by *Genewiz*, North Brunswick, NJ. Recombinant HA of H5VN04 was produced as described [1]. A/California/04/2009 (H1CA0409) and A/Singapore/1/57 (H2SIN57) recombinant HAs were supplied by Biodefense and Emerging Infections Research Resources Repository (BEI Resources).

### Generation of antibody variants

IGHV1-69*01 germline V-segment was synthesized by Geneart (Regensburg, Germany). The germline variant of VH1-69/F10 was constructed by ligating the IGHV1-69*01 gene (NcoI 5′, BssHII 3′) with F10 gene segment that included the CDR-H3+light chain (BssHII 5′ NotI 3′) into the pET22b vector, which was digested between NcoI-NotI. The various other VH1-69/F10 variants, HV1-69-sBnAbs derivatives of F10, A66, G17, D8, CR6261, and CR6331, as well as the F10 and A66 CDR-H2 variants were constructed using QuikChange Lightning Site-Directed Mutagenesis Kit (Strategene).

### Expression and purification of scFv

The scFv antibody sequences were cloned into the bacterial expression vector pET22b with an in-frame fusion of streptactin tag at the carboxy-terminus end. Plasmids were transformed into the expression BL21 (DE3) strain and the scFvs were produced by using the overnight express medium [52] according to the manufacturer protocol (Novagen). The scFvs were purified from clear bacterial cell lysates using the high-bind sepharose streptactin beads.

### Kinetic studies

Surface plasmon resonance (SPR) analysis was utilized for all kinetic measurements with a Biacore T100. For H5 binding kinetic studies, carboxyl terminus histidine tagged-H5 [1] was captured on a NTA-Ni+ activated chip. After stabilization period of 300 sec, the scFv in question was injected by using the single cycle kinetics function. Mobile phase contained HBS-P supplemented with 50 µM EDTA. Chip regeneration was carried out with two pulses of 0.3 M EDTA followed by injection of Nickel 50 µM of Ni^+^ solution.

### Panning of the phage display libraries

Panning of the phage display libraries was performed by standard immunotube approach [1].

### Binding and F10-epitope mapping competition assays of phage-Abs to HA antigens as determined by MSD-based ELISA assays

The Sector Imager 2400 from Meso Scale Discovery (MSD, Rockville, MD) is utilized for interrogating the binding activities between antibody and their respective antigens based on the manufacturer's instructions. Testing of purified fd-tet derived VH1-69/F10 phage-Ab variants was carried out by spot coating 6.25 ng of purified H5VN04 HA antigens onto 384-well high-bind MSD plates followed by incubation with serially diluted phage-Ab prep in triplicates. For F10-epitope competition assay with purified anti-H5VN04/H1CA0409 phage-Abs, the phage-Abs were added in duplicates to a plate precoated with purified HA from H5VN04 or H1CA0409 or H2SIN57, and blocked with F10-scFv or an irrelevant control scFv. Phage-Ab binding was detected with Sulfo-tagged anti-M13 mAb and assayed with a MSD Sector Imager 2400.

### Phage-Ab mediated neutralization assay

Phage-Ab mediated neutralization assay with H5V04 or H1PR8 pseudotyped luciferase-reporter lentiviral particles was performed according to previous published protocol [1] using purified phage-Abs at the concentration of 1.07e13 phage particles per ml.

### B-cell activation induced by BCR cross-linking

B-cell activation induced by BCR cross-linking was performed according to the study of Hoot et al [53].

### Binding of scFvs to H5VN04 as determined by standard ELISA assay

Standard ELISA assay was used to detect binding of the scFvs to H5VN04. Briefly, 2 µg/ml of H5VN04 was coated onto 384-well plates. Upon blocking with 2%BSA, purified strep-tagged scFvs were added onto the H5-coated plates and the binding was detected with Strep-Tactin-HRP mAb conjugate (IBA, GMBH) using PolarStar at 450 nm.

### Circular dichroism measurements

Circular dichroism measurements were performed with Aviv circular dichroism spectrometer model 202 (Aviv, Lakewood, NJ). PBS was served as a blank buffer control. The experimental conditions of the CD measurements were: averaging time −5 s; wavelength steps −1 nm; range from 195 to 255 nm; and temperature 25°C during the runs.

### Structural analysis and modeling

Molecular graphics and analyses were performed using PyMOL Molecular Graphics System, Version 1.5.0.4 Schrödinger, LLC, and by using the UCSF Chimera package [54] (http://www.cgl.ucsf.edu/chimera). Chimera is developed by the Resource for Biocomputing, Visualization, and Informatics at the University of California, San Francisco (supported by NIGMS P41-GM103311). The in-silico mutagenesis modeling was preformed according to the study of Fahmy *et al* [55].

### CDR definition and antibody numbering scheme

Our approach for defining the CDR domains was based on the IMGT definitions. However, for clarity reason we have decided to use the KABAT numbering system. In addition although not formally acknowledged, we have also defined a CDR-H4 domain as detailed in supplementary **Figure S2 in Text S1**.

### Data assembly and statistical analysis

HV1-69-sBnAb sequences were obtained through the NCBI website or published patents with the exception of the Ab named Kashyap1. Kashyap et al have published 61 clonally related heterosubtypic Abs in their 2008 study [2]. From the respective patent we have chosen the sequence of the HV1-69-sBnAb designated Ab1, which is claimed to be a heterosubtypic neutralizer. The reference dataset of functional *IGHV1-69* 51p1-allele germline based Abs was constructed using the Ig Blast website (www.ncbi.nlm.nih.gov/igblast/retrieveig.html ). Default parameters were kept for the categories of length and identity, synthetic Ab sequences were excluded, and in the germline gene name category the *IGHV1-69* 51p1 allele group gene were entered as IGHV1-69*01, 03, 05, 06, 07, 12 and *13*.

The retrieved 7 datasets were compiled into one 51p1 allele based Ab dataset and duplicated sequences were removed. In order to obtain a dataset characterized by Abs that start with first V-segment codon of Q1 (C.A.G) and do not surpass S113 (the last amino acid of the J-segment), the dataset was mapped to the reference IGHV1-69*01 gene, which allowed to crop Ab sequences that start with Q1 and then the cropped dataset was mapped again against a consensus J-segment (WGQGTLVTVSS) allowing the deletion of nucleotide sequences that go beyond S113 from the dataset. To facilitate the removal of clonally related Abs from the dataset, a CDR-H3 sub-alignment (C92-to-W103) was extracted and a sequence similarity matrix was organized by the name of the study. Studies found to be composed of identical CDR-H3 sequences (100% sequence identity) were taken out of the dataset. The resultant dataset was further cleaned by removal of sequence characterized by ambiguous nucleotide notations and of the studies detailed in **Table S1 in Text S1**. The entire dataset was translated, and was deleted of duplicated V-segments.

### Identification of unique amino acid substitutions in the HV1-69-sBnAb dataset

Using the UGENE software a matrix of amino acid substitutions was generated for the HV1-69-sBnAb and for the reference *IGHV1-69*-Ab datasets. A two-step method was used to identify distinctive amino acid substitutions associated with the HV1-69-sBnAb dataset. First, a Fisher's exact test was used to compare the distribution of amino acid substitutions at each position within the V-segment in the HV1-69-Abs dataset with that in the *IGHV1-69*-Ab reference dataset. Next, for germline positions where a significant statistical difference was found (P<0.05), another set of Fisher's exact tests were performed to compared the frequency of single amino acid substitutions. For the comparisons of individual substitution pattern at a given position, Bonferroni adjusted P-values were used to determine statistical significance in order to maintain an overall Type I error rate of 0.05 or less at each V-segment position.

### Identification of significantly enriched residues post phage-Ab selection

In order to study which of the non-germline amino acids in the semi-synthetic library were significantly enriched post H5VN04 and H1CA0409 selections (**Figure 4C** **and Figure S4B, E in Text S1**), and which CDR-H3 amino acids were enriched in the anti-H5VN04 heterosubtypic phage-Ab pool (**Figure S4C**) a matrix of amino acid substitutions was generated as described above. The hypothesis testing procedure was also performed as described above, except that chi-squared test was used to compare the distribution of amino acid substitution at the diversified positions against their respective positions in the design scheme (**Figure S3 in Text S1**). P-values were obtained by Monte-Carlo simulations, as chi-square approximation may not be appropriate with small sample sizes.

## Supporting Information

Text S1 The Supporting Text S1 file includes: Supporting Figure S1, which details the immunogenetic analysis of HV1-69-sBnAbs; Supporting Figure S2, which details how the CDR-H4 loop was defined; Supporting Figure S3, which describes the design scheme of the semi-synthetic *IGHV1-69* Ab library; Supporting Figure S4, which details the isolated anti-H5VN04 and anti-H1CA0409 phage-Ab pools; Supporting Figure S5, which describes the structural role of the HV1-69-sBnAbs distinctive amino acid substitutions in positions 52 and 52a; Table S1, which details studies reporting on the isolation of HV1-69-sBnAbs; Supporting Text, which details the design principles of the semi-synthetic *IGHV1-69* Ab library.
(DOCX)
