# Supplementary material for: Molecular Signatures of Hemagglutinin Stem-Directed Heterosubtypic Human Neutralizing Antibodies against Influenza A Viruses
Source: PLoS Pathog. 2014 May 1;10(5):e1004103. doi: 10.1371/journal.ppat.1004103 (PMC4006906; doi:10.1371/journal.ppat.1004103)
Supplement: Text S1 — The Supporting Text S1 file includes: Supporting Figure S1, which details the immunogenetic analysis of HV1-69-sBnAbs; Supporting Figure S2, which details how the CDR-H4 loop was defined; Supporting Figure S3, which describes the design scheme of the semi-synthetic IGHV1-69 Ab library; Supporting Figure S4, which details the isolated anti-H5VN04 and anti-H1CA0409 phage-Ab pools; Supporting Figure S5, which describes the structural role of the HV1-69-sBnAbs distinctive amino acid substitutions in positions 52 and 52a; Table S1, which details studies reporting on the isolation of HV1-69-sBnAbs; Supporting Text, which details the design principles of the semi-synthetic IGHV1-69 Ab library. (DOCX) [file ppat.1004103.s001.docx]

**Supporting Text 1**

A

D

**CDR-H1**

**CDR-H2**

**CDR-H4**

**71 73**

**51p1**

**33**

**54 56**

**85**

**hv1263**


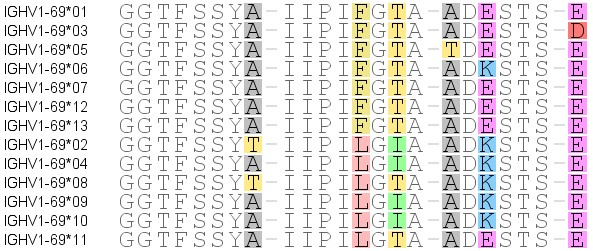


C

B

**Figure S1. Immunogenetic analysis of HV1-69-sBnAbs.**

HV1-69-sBnAbs were analyzed for **A)** V-segment allele usage (n = 38), with panel **B)** showing the 13 known *IGHV1-69* alleles and their classification into the 51p1 and hv1263 allele groups. Panel **C)** shows D-segment usage (n = 36), and panel **D)** shows J-segment usage (n =37).

B

AS


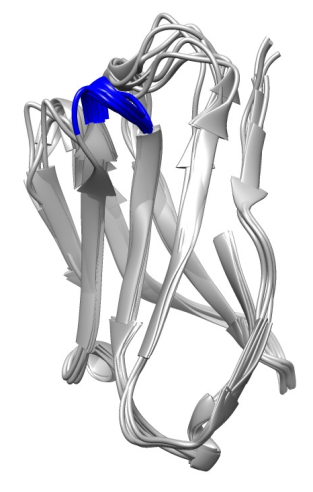


CDR-H2

CDR-H1

CDR-H4

**723**

**776**3

**Figure S2. Defining the CDR-H4 loop in *IGHV1-69* 51p1 allele based Abs.**

The existence of a CDR-H4 loop, or hypervariable loop-4 (HV4), has been suggested by several studies [[1](#_ENREF_1),[2](#_ENREF_2)]. However, no formal definition has been given to this loop. In this study the approach for defining the CDR-H4 loop was based on structural alignment, and by studying the overall nucleotide substitution frequency of the structurally defined CDR-H4 region as it appears in the reference *IGHV1-69*-Ab 51p1 allele based dataset (sees Methods for more details). **(A)** Structural alignment was performed for 7 non-antigen complexed *IGHV1-69* 51p1 allele related Abs: E51, 47e, 412d, CR9114, 1-69/b3, CR6261 and N12-i2. With the exception of N12-i2 all other six Abs were characterized by a loop that starts with position 73 and ends with position 76. Accordingly, the germline CDR-H4 loop sequence is defined as E.S.T.S. **(B)** The *IGHV1-69*-Ab 51p1 allele based reference dataset was analyzed for non-germline nucleotide substitution frequencies. The red line shows the mean of non-germline nucleotide substitution frequency observed for FR regions and dashed lines point to the CDR areas. The analysis of variance (ANOVA) shows (**B — inset**) that the mean of non-germline nucleotide substitution frequency of the CDR-H4 area is significantly higher than that of the FRs (P = 0.02), but is not significantly different than that of the CDR-H1+H2 (P = 0.27).

**c**

**d**

A

B

**Figure S3. The design principles of the *IGHV1-69* synthetic library.**

**A**) Diversification plan of the V and J segments. In red are amino acids that were elevated beyond their natural observed frequency. **B**) Diversification scheme for the CDR-H3 domain, which was based on the natural frequency and diversity observed in a reference CDR-H3 alignment made from *IGHV1-69* 51p1allele based Abs (n =1217).

A

B

|  | Library design scheme | Anti-H5VN04 phage-Abs | Ratio | *P^a^* |
| --- | --- | --- | --- | --- |
| HV1-69-sBnAb distinctive amino acid substitutions* | | | | |
| CDR-H1 |  |  |  |  |
| G27V | 10% | 39% | 3.9 | 0.0005 |
| CDR-H2 |  |  |  |  |
| Ser52 | 10% | 81% | 8.1 | 0.0035 |
| Non-HV1-69-sBnAb distinctive amino acid substitutions | | | | |
| CDR-H2 |  |  |  |  |
| I53M | 11% | 36% | 3.4 | 0.007 |
| CDR-H4 |  |  |  |  |
| S76N | 11% | 31% | 2.8 | 0.004 |
| CDR-H3 |  |  |  |  |
| G97 | 19% | 53% | 2.8 | 0.01 |
| Y98 | 11% | 78% | 7.1 | 0.01 |
| Y99 | 12% | 33% | 2.7 | 0.03 |
| P100 | 4% | 34% | 8.1 | 0.01 |
| G100B | 13% | 43% | 3.3 | 0.01 |
| a - P-values were obtained by Monte-Carlo simulations.  *- See Figure 2B. | | |  |  |

C

|  | Library deign scheme | Heterosubtypic subset CDR-H3 (n = 16) | Non-heterosubtypic subset CDR-H3 (n = 8) |
| --- | --- | --- | --- |
| Y98 | 11% | 100% (16) * | 100% (8) * |
| Y99 | 12% | 44%*(7)* | 0% |
| P100 | 4% | 38%* (6)* | 38%(3) † |
| G100B | 13% | 63%*(10)* | 50% (4) † |
| *- Significant change from the library design scheme. P < 0.05 as determined by Monte-Carlo simulations.  † Non-significant change from the library design scheme. | | | |

D

E

D

|  | Library design scheme | Anti-H1CA0409 phage-Abs | Ratio | *P^a^* |
| --- | --- | --- | --- | --- |
| HV1-69-sBnAb distinctive amino acid substitutions* | | | | |
| CDR-H1 |  |  |  |  |
| G27V | 10% | 33% | 3.3 | 0.002 |
| CDR-H2 |  |  |  |  |
| Ser52 | 10% | 57% | 5.7 | 0.0035 |
| Non-HV1-69-sBnAb distinctive amino acid substitutions | | | | |
| CDR-H4 |  |  |  |  |
| S76N | 11% | 30% | 2.7 | 0.014 |
| CDR-H3 |  |  |  |  |
| G97 | 19% | 60% | 3.1 | 0.01 |
| Y98 | 11% | 53% | 4.9 | 0.01 |
| a - P-values were obtained by Monte-Carlo simulations.  *- See Figure 2B. | | |  |  |

**Figure S4. CDR sequences and binding characteristics of the anti-H5VN04 and anti-H1CA0409 phage-Ab pools**

**A)** CDR sequence alignment of the anti-H5VN04 phage-Ab pool was ordered based on heterosubtypic binding activity as tested against H1CA0409 and H2SIN57. Highlights point to the statistically significant enriched residues post selection as discovered by Chi square test in **B)**. **C)** Heterosubtypic (n=16) and non-heterosubtypic (n=8) anti-H5VN04 phage-Abs that are characterized by Ser52/Tyr98 were analyzed separately by Chi square test for the frequency of enriched amino acids in CDR-H3. **D)** CDR sequence alignment of the anti-H1CA0409 phage-Ab pool was ordered based on heterosubtypic binding activity as tested against H5VN04 and H2SIN57. Highlights point to the statistically significant enriched residues post selection as discovered by Chi square test in **E**.Tyr99 was also highlighted. Red circles point to the two phage-Abs which did not bind to the stem domain of H1CA0409. Blue circles point to common phage-Ab clones discovered independently from both panning campaigns.

B

A

**Ile51**

**Ser52**

**Pro52a**

**Met53**

**Phe54**

**Gly55**

**Thr56**

**Pro57**


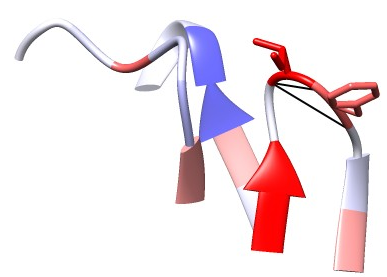


**Gly55**

**GLY52a**

**CDR-H2**

**CDR-H1**

| **Structural model of F10 wt CDR-H2 loop** | |
| --- | --- |
| **CDR-H2 residue** | **#VDW contacts** |
| Met53 | 1 |
| Phe54 | 3 |
| Gly55 | 1 |
| Thr56 | 2 |
| **Total VDW contacts** | **7** |

| **Structural model of 1-69.b3 P52aG variant** | |
| --- | --- |
| **CDR-H2 residue** | **#VDW contacts** |
| **CDR-H2** |  |
| Phe54 | 1 |
| Gly55 | 1 |
| **Total VDW contacts** | **2** |


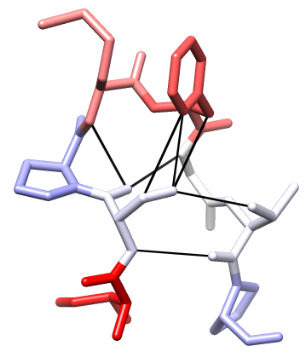


**-4.5 ARG**

**-0.4 GLY**

**2.8 PHE**

**4.5 ILE**

| **Structural model of**  **1-69.b3 wt** | |
| --- | --- |
| **CDR-H2 residue** | **#VDW contacts** |
| **CDR-H1** |  |
| Phe29 | 2 |
| Tyr32 | 1 |
| Ala33 | 1 |
| **CDR-H2** |  |
| Ile51 | 2 |
| Phe54 | 2 |
| Gly55 | 3 |
| **FR3** |  |
| Ala71 | 1 |
| **CDR-H4** |  |
| Glu73 | 1 |
| **Total VDW contacts** | **13** |

| **Structural model of F10 S52I CDR-H2 loop** | |
| --- | --- |
| **CDR-H2 residue** | **#VDW contacts** |
| Ile51 | 7 |
| Pro52a | 1 |
| Met53 | 5 |
| Phe54 | 7 |
| Gly55 | 1 |
| Thr56 | 3 |
| **Total VDW contacts** | **24** |

**Ile51**

**Ile52**

**Pro52a**

**Met53**

**Phe54**

**Gly55**

**Thr56**

**Pro57**


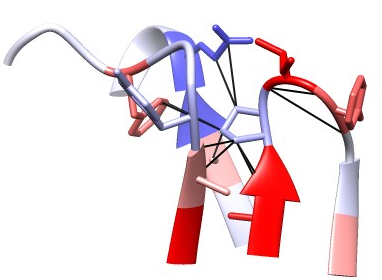


**CDR-H1**

**CDR-H2**

**CDR-H2**

**Phe29**

**Glu73**

**Pro52a**


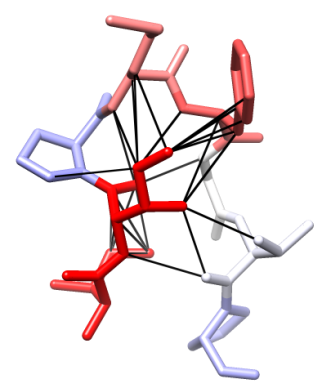


**Figure S5. Understanding the structural role of the HV1-69-sBnAbs distinctive AA substitutions in positions 52 and 52a.**

**A)** In the model of non-HA complexed F10, VDW contacts were analyzed for Ser52 against other CDR-H2 loop residues (***upper panel*)**. Ser52 was in-silico mutagenized [[3](#_ENREF_3)] to Ile52 to show the occurrence of a much higher number of VDW contacts (***lower panel*)**. **B)** ***Upper panel*** – Pro52a in 1-69/B3 (mAb characterized by a non-mutated *IGHV1-69*01* V-segment [[4](#_ENREF_4)]) was in-silico mutagenized to Gly52A to show the occurrence of minimal number of VDW contacts as compared to the germline Pro52a as shown in the ***lower panel***. Coloring scheme is based on the Kyte-Doolittle hydrophobic scale.

**Supplemental Table**

| **Study†** | **Panning method** | **Panning target** | **# of Positive clones^a^** | **Frequency of**  ***IGHV1-69* Abs** | **# of anti-stem binding clones** | **Frequency of**  ***IGHV1-69* based anti-stem Abs** |
| --- | --- | --- | --- | --- | --- | --- |
| Kashyap et al.  (2008) | Phage display library generated from bone marrow B cells of H5N1 Turkish survivors. | H5N1 | The study reports 61 clonally related *IGHV1-69* based sequences. However, the full binding properties of isolated Abs was not reported. | | | |
| Throsby et al.  (2008) | Phage display library generated from IgM+ memory B cells of H5N1 vaccinated donors. | H5N1 | 43 | 15/43 | 13 | 12/13 (92%) |
| Sui et al.  (2009) | Naïve human antibody phage display library. | H5N1 | 6 | 5/6 | 6 | 5/6 (83%) |
| Corti et al. (2010)+ | Panning B-cells from donors vaccinated with the seasonal influenza vaccine. | H5N1/H1N1 | 20 | 14/20 | 19 | 14/19 (74%) |
| Wrammert et al. (2011)+ | Panning plasmablasts from 2009 pandemic H1N1 infected donors. | H1N1 | 15 | 4/15 | 5 | 4/5 (80%) |
| Thomson et al. (2012)+ | Panning B-cells and plasmablasts from 2009 pandemic H1N1 infected and vaccinated donors. | H1N1 | 48 | 25/48 | 7* | 7/7 (100%) |
| Li et al. (2012) + | Panning plasmablasts from 2009 pandemic H1N1 vaccinated donors. | H1N1 | 28 | 1 | 3 | 1/3 (33%) |
| a — number represents distinctive VH sequence.  *Not all positive clones were epitope mapped.  † Dreyfus et al. (2012) has reported the isolation of one *IGHV1-69* germline based anti-stem Ab.  + Naturally paired VH and VL chains show biased *IGHV1-69* usage and promiscuous use of VL, similarly to sBnAbs isolated from Ab-phage display libraries. | | | | | | |

**Table S1. Summary of various studies that have analyzed antibody responses towards hemagglutinin of group 1 influenza A subtypes.**

**Supporting text:**

**The design principles of the semi-synthetic *IGHV1-69* Ab library.**

The main goal of the *semi-synthetic IGHV1-69 Ab library* diversification scheme was to obtain an Ab library characterized by the V-segment molecular determinants associated with HV1-69-sBnAbs, while maintaining overall low V-segment amino acid substitution frequency. At the time the library was designed our knowledge of V-segment molecular determinants of *IGHV1-69*-sBnAbs was based on the structural analysis of F10 and CR6261 solved structures, and from an alignment made of HV1-69-sBnAbs reported by Thorsby et al., (2008) [[5](#_ENREF_5)] Sui et al.,[[6](#_ENREF_6)] (2009) and Corti et al (2010)[[7](#_ENREF_7)]. This analysis has suggested that the amino acid substitutions of CDR-H1: Val27, Ile28, Pro29, Arg30; CDR-H2: Ser52, Gly52a, Ala52a; and CDR-H4 Glu73 and Phe74 were distinctly associated with HV1-69-sBnAbs. It was therefore decided that these amino acid substitutions should be included in the library diversification scheme at the relatively elevated frequencies of 5-to-10% (**Figure S3A**).

In order to explore if generation of HV1-69-sBnAbs based on amino acid substitutions occurring in positions CDR-H2 52 and 52a is restricted to SER52, GLY52a and ALA52a, these positions were also diversified with naturally occurring amino acid substitutions using frequencies that were acquired from a CDR-H2 alignment composed of *IGHV1-69* 51p1 allele based Abs that were devoid of germline CDR-H2 sequences (n = 800). In addition, CDR-H2 position 53 was also diversified according to this alignment for the reason that HV1-69-sBnAbs characterized by I52aG were always found to be accompanied by mutated positions 53 (either I53V or I53M) suggesting the existence of structural dependency. Likewise, since it was noticed that several HV1-69-sBnAbs are highly diversified in the surrounding CDR-H4 domain area (**Figure S2**), we decided that positions of 73, 74, 76 and 78 will be diversified with naturally occurring amino acid substitutions at a frequencies that were observed in a 73-to-78 alignment made from *IGHV1-69* 51p1 allele based Abs (n = 1477). To insure that the library would maintain “germline” characteristics, the frequency of the respective germline amino acids in each of the diversified positions was maintained at high frequencies of 72%-to-95%.

For the CDR-H3 domain, the strategy was to have an equal presentation of CDR-H3 lengths of 5, 7, 9, 10, 11, 12, 13, and 15, whereby diversification scheme (**Figure S3B**) was based on the natural diversity observed in alignment composed of 1217 non-duplicated *IGHV1-69* 51p1 allele based Abs. In order to avoid skewing of the observed CDR-H3 frequencies due to residues associated with J-segments, the CDR-H3 sequences were aligned and deleted of amino acids post position 100 (which is Phe in IGHJ1-5). Additionally, the CDR-H3 sequences were also omitted of IGHJ6’s quintuple tyrosine residues (YYYYYGMDVWGQGTTVTVSS) as these were found to distort the amino acid frequency and diversity in the alignment. The design of the J-segment was based on a consensus sequence of: FDVWGQGTLVTVSS, in which F and V were diversified according to the frequency scheme presented in **Figure S3A.**

The library light chains were obtained from the previously constructed Mehta I/II naïve human light chain Ab libraries and were linked to the VH synthetic library via a (GLY_3_SER)_4_ linker. Synthesis of the *IGHV1-69* library was performed by Morphosys GmBH based on the technology developed by Sloning GmBH. Generation of fd-tet scFv phage display library was performed as described previously [[8](#_ENREF_8)]. Phage library size consisted of 7.7X10^8^ members. Randomly sequencing of 164 Ab library members validated a similar diversification scheme (data not shown) and confirmed low V-segment amino acid diversity with a mean of 1.9±1.1 amino acid substitutions per V-segment.

**References:**

1. Fanning SW, Horn JR (2011) An anti-hapten camelid antibody reveals a cryptic binding site with significant energetic contributions from a nonhypervariable loop. Protein Sci 20: 1196-1207.

2. Briney BS, Willis JR, Crowe JE, Jr. (2012) Location and length distribution of somatic hypermutation-associated DNA insertions and deletions reveals regions of antibody structural plasticity. Genes Immun 13: 523-529.

3. Fahmy A, Wagner G (2011) Optimization of van der Waals energy for protein side-chain placement and design. Biophys J 101: 1690-1698.

4. Almagro JC, Beavers MP, Hernandez-Guzman F, Maier J, Shaulsky J, et al. (2011) Antibody modeling assessment. Proteins 79: 3050-3066.

5. Throsby M, van den Brink E, Jongeneelen M, Poon LL, Alard P, et al. (2008) Heterosubtypic neutralizing monoclonal antibodies cross-protective against H5N1 and H1N1 recovered from human IgM+ memory B cells. PLoS One 3: e3942.

6. Sui J, Hwang WC, Perez S, Wei G, Aird D, et al. (2009) Structural and functional bases for broad-spectrum neutralization of avian and human influenza A viruses. Nat Struct Mol Biol 16: 265-273.

7. Corti D, Suguitan AL, Jr., Pinna D, Silacci C, Fernandez-Rodriguez BM, et al. (2010) Heterosubtypic neutralizing antibodies are produced by individuals immunized with a seasonal influenza vaccine. J Clin Invest 120: 1663-1673.

8. O'Connell D, Becerril B, Roy-Burman A, Daws M, Marks JD (2002) Phage versus phagemid libraries for generation of human monoclonal antibodies. J Mol Biol 321: 49-56.
